# Comparing the effects of self-selected music versus predetermined music on patient anxiety prior to gynaecological surgery: a study protocol for a randomised controlled trial

**DOI:** 10.1186/s13063-018-3093-6

**Published:** 2019-01-07

**Authors:** Tiphaine Petot, Nicolas Bouscaren, Olivier Maillard, Laetitia Huiart, Malik Boukerrou, Danielle Reynaud

**Affiliations:** 1 CHU de La Réunion, INSERM, CIC1410, 97410 Saint Pierre, France; 2 INSERM, IRD, SESSTIM, Sciences Économiques et Sociales de la Santé et Traitement de l’Information Médicale, Aix Marseille University, 13006 Marseille, France; 3 Service de Gynécologie et d’Obstétrique, CHU de La Réunion, 97410 Saint Pierre, France; 4 INSERM, EA7388 « Centre d’Etudes Périnatales de l’Océan Indien » (CEPOI), 97410 Saint Pierre, France

**Keywords:** Anxiety, Music therapy, Gynaecological surgery, Alternative and complementary therapy

## Abstract

**Background:**

Patients undergoing gynaecological surgery are known to experience anxiety. While the use of music selected by patients based on their personal taste has hardly been evaluated, a recent study suggests that musical preferences significantly alter the anxiolytic and relaxing effects of music. Our study aims to determine whether self-selected music decreases patient anxiety prior to gynaecological surgery, as compared with predetermined music from a software programme such as MUSIC CARE®.

**Methods:**

The study will consist of a clinical trial comparing the effects of self-selected music versus predetermined music on patient anxiety prior to gynaecological surgery. A minimum of 170 patients will be randomised in a 1:1 ratio. Inclusion criteria will be: women aged 18–55 years, awaiting scheduled gynaecological surgery under general/local anaesthesia or under sedation; having created a personal 20-min playlist; and not having received anxiolytic drugs prior to surgery. The primary outcome will be the difference between the preoperative anxiety score taken 15 to 20 min before the music-listening session and the preoperative anxiety score taken shortly after the session, as measured with the STAI for state anxiety.

**Discussion:**

This study should help to identify more effective non-medical treatments for preoperative anxiety, as well as to adapt music therapy to the cultural context of patients.

**Trial registration:**

ClinicalTrials.gov, ID: NCT03226834. Registered on 24 July 2017.

## Background

Anxiety is a physiological and psychological state characterised by somatic, emotional, cognitive, and behavioural components. Surgical procedures and preoperative wait are anxiety-provoking situations [1]*.* It is estimated that the incidence of preoperative anxiety ranges from 60 to 80% [2, 3]. A high level of anxiety can lead to an increase in heart and respiratory rates, blood pressure, cortisol levels, and feelings of fear and uncertainty [4, 5]. It can also hinder the wound healing and immune system response, increasing risks of infection and postoperative complications [1]. Stress and anxiety are especially high among patients who have learned that they must undergo gynaecological surgery, as the latter comes with a high risk of partial or complete removal of one or more organs associated with motherhood or femininity [6].

In preoperative settings, it is common practice to prophylactically administer sedative or anxiolytic drugs in order to reduce patient anxiety. Yet, these treatments often cause side effects like drowsiness and breathing difficulties, and they can interact with anaesthetic agents by prolonging awakening duration and hospital stay [7]. In view of this, non-medical strategies such as music therapy, hypnosis, acupressure, and sophrology are increasingly used to reduce preoperative anxiety. In parallel, research on music therapy has grown exponentially over the last 20 years [1, 8]. Randomised controlled trials (RCTs) have highlighted the anxiolytic effects of music therapy in surgery [9–12], cardiology [13–15], and oncology [16, 17]. Moreover, a 2013 systematic literature review has presented a meta-analysis of 26 trials covering a total of 2051 participants, which suggests that listening to music has beneficial effects on preoperative anxiety compared with routine care alone [1]. In 2018, another systemic review examined the effectiveness of music as an adjunct treatment in anxiety for patients undergoing hip and knee surgery and showed that music improved anxiety in six of the ten studies [18].

Nevertheless, most published RCTs evaluate the effects of music therapy on preoperative anxiety by comparing patients who listen to researcher-selected or predetermined music (from a software programme such as MUSIC CARE®) with patients who listen to no music at all. The effects of self-selected music on patient anxiety have hardly been investigated [19, 20]. One exception is a recent study on the anxiolytic and relaxing effects of music, which has highlighted the importance of preference and familiarity in eliciting the relaxation response [21].

Perceptions of music styles, rhythms, and sounds are known to vary depending on cultural background, ethnicity, and personal experience. However, music therapy in preoperative settings currently relies on software programmes that provide pre-selected, standardised music playlists. This is the case of MUSIC CARE®, the music therapy programme that has been in use in our gynaecology department for the past 10 months. Though the efficacy of MUSIC CARE® has been demonstrated, the playlists it provides (jazz, classical music, world music, etc.) are not totally adapted to the specific cultural contexts of users. For example, in Reunion Island, a French overseas territory with a multiethnic and multicultural population, two popular music styles are *Séga* and *Maloya*. This type of music is not included in MUSIC CARE® software.

Our study seeks to fill this gap by letting patients create their own, culturally adapted, music playlist. Our assumption is that a self-selected playlist composed of patients’ preferred songs will have beneficial effects on preoperative anxiety.

The aim of our study is to determine whether listening to self-selected music decreases patient anxiety as compared with predetermined music from a software programme such as MUSIC CARE®. To do this, we will conduct a parallel, superiority, RCT comparing the effects of self-selected music versus predetermined music on patient anxiety prior to gynaecological surgery.

## Methods: participants, interventions, and outcomes

### Study setting

This study will be a monocentric, parallel, superiority, RCT conducted in the Gynaecological Surgery Department of Reunion Island Hospital. Patients will be randomly allocated to one of two groups: one group will listen to self-selected music and the other to predetermined music.

### Eligibility criteria

#### Inclusion criteria

Inclusion criteria will be as follows: women aged 18 to 55 years; awaiting scheduled gynaecological surgery under general/local anaesthesia or under sedation; not having received anxiolytic drugs prior to surgery; having a good understanding of French; knowing how to read and write; having signed an informed consent form. To be included in the study, patients will need to have created a personal 20-min playlist for the study.

#### Exclusion criteria

Exclusion criteria will be as follows: having severe hearing loss or speech impairment; being deprived of liberty by judicial or administrative decision; being under trusteeship or guardianship; suffering from dementia; having a history of anxiety or depression; suffering from neuromotor disabilities; undergoing emergency or ambulatory surgery.

### Intervention

#### Intervention group

Patients will listen to the music playlist they will have created at home based on their personal taste. The equipment used will vary according to the patient: a smartphone, a touchpad, a computer, or a CD player.

#### Control group

Patients will listen to a predetermined music playlist that they will have chosen among those available on the MUSIC CARE® software. Patients can choose among 25 playlists, including reverie, Cuban night, guitar ballad, film scores, folk guitar, etc.

MUSIC CARE® is a non-drug alternative for the management of anxiety and pain that uses a form of music therapy known as the ‘U sequence’. The latter is based on the hypno-analgesia principle, whereby progressive variations in tempo, rhythm, tune, and harmony bring patients into a state of deep psycho-muscular relaxation before the awakening phase. The objective of MUSIC CARE® is to promote wellbeing and to induce a relaxed state in patients by loosening their muscles and reducing their level of anxiety or pain.

The U sequence is only for the control group and is composed of three phases:

1. Sleep-inducing phase (stimulating rhythms), which corresponds to the patient’s current state of consciousness
2. Relaxation phase (slow rhythms)
3. Awakening phase (moderate rhythms), which signals the end of the session

#### For each group

An inclusion visit will take place the day before surgery. During this visit, the investigator will provide detailed information on the study and answer the patient’s questions concerning the objectives, constraints, foreseeable risks, and perceived benefits of the study. The investigator will also be responsible for obtaining written informed consent from the patient.

**Fig. 1:**
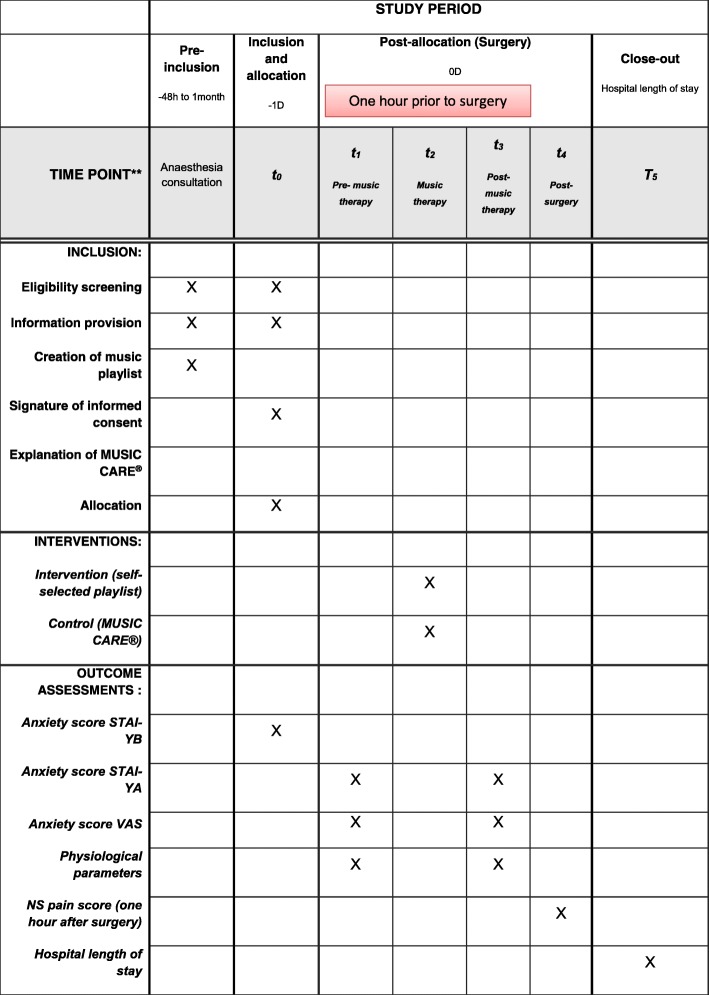
Standard Protocol Items: Recommendations for Interventional Trials (SPIRIT) Figure: schedule of enrolment, intervention, and assessments

**Fig. 2:**
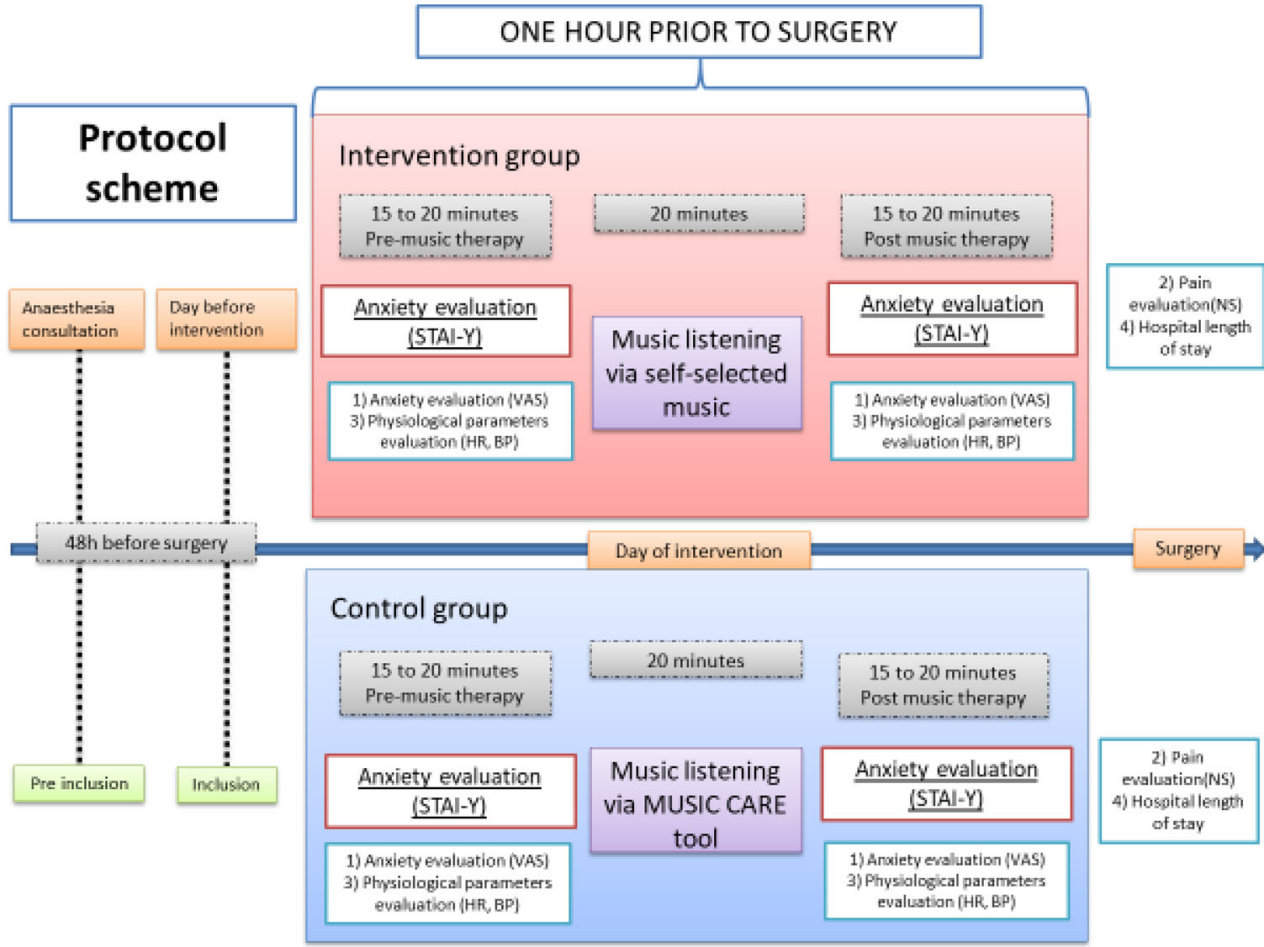
Diagram to show the timing when each outcome take place

During the inclusion visit, a department nurse or care provider with training in MUSIC CARE® will explain the software to the study participant. Each patient will be asked to choose a predetermined music playlist from those available on the MUSIC CARE® software. The following day (i.e. the day of surgery), participants in the control group will be asked to listen to this music playlist, while those in the intervention group will be asked to listen to the music playlist they will have created at home. There will be only one music-listening session in the hour preceding surgery. The session duration will be 20 min. To ensure that patients do listen to a music playlist, a trained research team member will bring a MUSIC CARE® tablet to each patient, notify her of the group to which she has been allocated, and inform her that she can start listening to the music playlist on the support of her choice. After 20 min, the research team member will return to inform the patient that the session has ended and will recover the equipment. All the interventions for each group are summarised in Figs. 1 and 2. During the music-listening session, patients will be alone in their individual room, seated in an armchair or lying in the supine position. They will not be disturbed by anyone. The music will be played through headphones with disposable foam ear cushions to avoid nosocomial infection, and there will be no limit on volume levels. Patients will be reminded that they can listen only to the MUSIC CARE® playlist or the self-selected playlist (and to nothing else) during the session. The care team will be blinded to patient randomisation.

If a patient receives anxiolytic drugs the day before or the day of surgery, she will be withdrawn from the trial. All patients will receive routine care.

### Outcomes

The primary outcome will be the difference between the preoperative anxiety score taken 15 to 20 min before the music-listening session and the preoperative anxiety score taken shortly after the session, as measured with the STAI (State-Trait Anxiety Inventory) for state anxiety [22]. We will use the mean change score from baseline to post music intervention to compare groups. The STAI is a validated and widely used questionnaire for measuring patient anxiety, in particular in the context of music therapy. This self-assessment questionnaire consists of 40 statements, each describing an attitude. It is composed of two separate sections of 20 statements each: The first section assesses trait anxiety and the second measures state anxiety. In each section, ten statements express a negative attitude (e.g. ‘I am worried’) and the other ten express a positive attitude (e.g. ‘I feel calm’). In the section measuring trait anxiety, participants answer each item to report the frequency of their feelings. All items are rated on a Likert-type 4-point scale ranging from ‘almost never’ to ‘sometimes’, ‘often’, and ‘almost always’. In the section measuring state anxiety, participants answer each item to report the intensity of their feelings in the preoperative situation. All items are rated on a Likert-type 4-point scale ranging from ‘not at all’ to ‘somewhat’, ‘moderately so’, and ‘very much so’. STAI results are obtained by adding the scores for negatively worded items and subtracting the scores for positively worded items. Total scores for trait and state anxiety range between 20 and 80: The higher the score, the more the patient is anxious for both types of anxiety. The time needed for the patient to take the test is between 10 and 15 min.

In our study, we will use the validated French version of the STAI (‘Inventaire d’Anxiété Situationnelle et de Trait d’Anxiété’ (IASTA)) [23]. Patients will be asked to fill the first section of the self-assessment questionnaire (trait anxiety) the day before surgery, during the inclusion visit. The day of surgery, they will be asked to fill the second section (state anxiety), which assesses ‘how the individual feels now, at this moment, in terms of concern, tension or nervousness’. A department nurse will administer the questionnaire 15 to 20 min before the start of the music-listening session, and will do so a second time shortly after the session has ended.

There will be four secondary outcomes in our study. The first will be the difference between the preoperative anxiety score before the music-listening session and the preoperative anxiety score after the session, as measured with the visual analogue scale (VAS). The VAS is a calibrated plastic stick that can be used horizontally or vertically. A slider moves along the plastic stick on the side presented to the patient, with the words ‘calm, not at all anxious’ appearing on one end of the stick and the words ‘extremely anxious’ appearing on the other. The patient must place the slider in the position that better describes their level of anxiety. On the other side of the stick is a graduation scale, which is seen only by the care provider. The latter reads the patient’s level of anxiety on a scale from 0 to 10 based on the slider position selected. Studies have shown that anxiety levels measured with the VAS are significantly correlated to those measured with the STAI questionnaire.

In our study, a department nurse will collect VAS values for each patient. Before administering the test, the nurse will explain to the patient how the scale works and will make sure that she understands the information. The test will be administered 15 to 20 min before the start of the music-listening session, and a second time shortly after the session has ended (i.e. after the patient has filled the second section of the STAI self-assessment questionnaire and before she goes to the operating room).

The second secondary outcome will be the difference in pain values in immediate post surgery between the intervention group (patients listening to self-selected music) and the control group (patients listening to predetermined music), as measured with a numerical scale (NS). This self-reported scale is administered by asking the patient to rate their level of pain from 0 (no pain) to 10 (worst pain). In our study, a nurse will measure NS values 30 min after the patient has returned from the operating room.

The third secondary outcome will be the difference in preoperative physiological parameters (heart rate and systolic/diastolic blood pressure) measured before and after the music-listening session. A nurse will record these parameters 15 to 20 min before the session, after she administers the STAI and VAS tests, and will do so again shortly after the session, after she administers the STAI and VAS tests a second time and before the patient goes to the operating room. Physiological parameters will be measured in the Gynaecological Surgery Department with an automatic electronic tensioning device (reference: CRITICARE EQUALITY Series 506DN Comfort plus®) consisting of an adult-size inflatable Velcro armband. The armband size will be adapted to the patient’s arm (different sizes are available in our department). The blood pressure of each patient will be taken at 7.00 a.m. The patient will be informed that she must remain calm in the room for at least 10 min before measurements are taken. Physiological parameters will be measured with the patient relaxed in a supine position, on a bare arm with the crook of the elbow placed at the level of the heart. When attaching the armband, the nurse will make sure that the measurement sensor faces the brachial artery. Blood pressure will not be measured on a perfused or injured arm, on an arm with an arteriovenous fistula, or on an arm that has undergone lymph-node dissection. Blood pressure will be recorded in millimetres of mercury and heart rate will be recorded in beats per min.

The fourth secondary outcome will be hospital length of stay from hospital admission to hospital discharge.

The assessment of each outcome and the timing are resume in the Figs. 1 and 2.

### Sample size

According to a meta-analysis published in 2013, the average preoperative STAI anxiety score reported in the literature is 43 ± 8. However, this score is 38 + 8 in patients who listen to music prior to surgery compared with patients who receive conventional care alone [1]. Our study hypothesis is that there will be a further decrease of 3 points in the intervention group compared with the control group. This difference will be considered clinically relevant. Furthermore, we posit an intra-individual correlation equal to 0.5 between the measure taken before the music-listening session and the measure taken after the session (conservative assumption). Under this hypothesis, the inclusion of 85 patients in each group (170 in total) will highlight a significant difference in anxiety reduction between the control group (6-point decrease) and the intervention group (9-point decrease), with an alpha level of 5% and a statistical power of 80%.

### Recruitment

The recruitment will take place in the Obstetrics and Gynaecology Department of the University Hospital. The pre-inclusion visit will be performed by physician during the anaesthesia visit between 48 h and 1 month before the inclusion visit corresponding to the day before hospitalisation for surgery. During this pre-inclusion visit, a screening of patients eligible for the study will be carried out. The physician will inform the patient and answer all her questions regarding the objective, the nature of the constraints, the foreseeable risks and the expected benefits of the research. If she wishes to participate, she is asked to create a playlist on the device of her choice, from her own collection to be brought in on the day of hospitalisation for surgery. Each week, 15 to 25 patients are planned for an anaesthesia consultation and may be eligible for inclusion.

## Methods: Assignment of interventions

Eligible patients will be randomised on the day of surgery using EnnovClinical®. Randomisation will be balanced in both groups, single-blinded, with a 1:1 allocation ratio, without stratification. The patient will know her allocation to the control group or to the intervention group, but the care-giver team will be unaware of it. The randomisation list will be established by the statistician from the methodological support department before the start of the research. The method used will be a randomised block approach, using the SAS® Proc Plan. We will build blocks of variable size. The investigator will perform the randomisation directly via the electronic case report form (eCRF) using the CS Random module of the Ennov Clinical® eCRF the day of surgery.

Routine care providers and care team members performing outcome assessment visits will be blinded to the patients’ group allocation. Only the research team will be aware of the group to which patients have been allocated. ‘Do Not Disturb’ signs will be placed on patient room doors during the listening session. Patients will be warned not to mention their group allocations to care providers.

## Methods: Collection, management, and analysis of data

### Data collection method

Data will be collected using an eCRF.

The database will be created by a data manager of the Methodology and Data Management Centre using EnnovClinical® software. Database access will be protected with a user name and password, and will include various safety levels according to role assigned. Research team members will enter the data.

Data-entry agents who are used to working on the eCRF will be responsible for entering the participants’ data. Data-entry and consistency checks will be carried out at the end of the inclusion period by a third party.

### Statistical methods

Qualitative variables will be expressed as absolute numbers and percentages with a confidence interval of 95%. Quantitative variables will be expressed as averages, standard deviations, and ranges if they are normally distributed, and as medians, interquartile ranges, and ranges if they are not. Total sample size will be reported for each analysis. A difference will be considered significant when the *p* critical value is lower than 5%.

#### Primary outcome assessment

Anxiety scores measured after the music-listening session will be compared between the two groups, using an analysis of covariance model (ANCOVA) adjusted to the group and to the anxiety score measured before the music-listening session. This method is recommended for analyses of RCTs that evaluate a variation between an initial value and a post-intervention value.

#### Secondary outcomes assessment

Comparisons of VAS values and physiological parameters between the two groups will be performed using the same method as will be used for the primary outcome. Comparisons of immediate post-surgical pain and hospital length of stay between the two groups will be performed using the Student’s *t* test or the Mann-Whitney *U* test, as appropriate.

An intention-to-treat and per-protocol analysis will be conducted. Subgroup analyses considering anxiety, pain, physiological parameters, and length of stay by type of surgery will be performed.

## Data monitoring

### Data monitoring

A clinical research associate (CRA) mandated by the study sponsor will visit the gynaecological surgery department twice during the study and once at the end. During these visits the CRA will verify: all consent forms; compliance with established protocol and procedures; quality of the data collected in the eCRF. The CRA will write a monitoring report after each visit. There will not be an independent body monitoring safety. Access to the database will be secured (access by personal ID and password) with different levels of security depending on the roles assigned. The data hosting is certified ISO 9001:2015. Ennov® hosts its customers’ applications in a data centre located in two mirror sites offering optimal security conditions. Ennov Clinical® complies with Food and Drug Administration (FDA) recommendations regarding Computerised Systems for Clinical Trials Management (‘Guidance for computerised systems used in clinical trials’) and electronic signature (‘21 CFR Part 11’) and international standards.

### Harms

Adverse effects and serious adverse effects will be identified according to standard criteria. They will be recorded in the eCRF and transmitted to the sponsor by the investigator. No serious adverse effects are expected.

All complications and adverse events will be recorded and analysed.

### Auditing

A person mandated by the sponsor may conduct an audit at any time during the study, independently of the investigating team.

## Ethics and dissemination

### Protocol amendments

A Steering Committee will decide on any relevant amendments to the protocol necessary for the continuation of the research. Any substantial modification, i.e. any modification likely to have a significant impact on the protection of persons, conditions of validity, on the results of the research, or on the methods of conducting the research, will be the subject of a written amendment which will be submitted to the sponsor: the latter must be obtained, prior to its implementation, including an assent from the Humans Ethics Committee.

### Consent

The investigating physician is responsible for obtaining the patient’s written informed consent.

### Access to data

Only investigators will have access to the final dataset.

### Dissemination policy

Any written or oral communication of the results of the research must be approved by the members of the Steering Committee. The publication of the main results will mention the name of the sponsor, all investigators who included or followed patients in the research, methodologists, biostatisticians and data managers who participated in the research. International writing and publication rules will be taken into account (The Uniform Requirements for Manuscripts of the ICMJE, April 2010).

## Discussion

This is a protocol for a randomised, single-blinded, controlled trial evaluating the effects of music therapy on patient anxiety. This trial is justified by the lack of knowledge on the effects of self-selected music versus predetermined music on patient anxiety prior to gynaecological surgery.

At present, music therapy software programmes offer predetermined, standardised playlists that fail to include many of the music styles that exist around the world. Our study will let patients create personal playlists composed of their favourite songs in order to highlight the role of preference and familiarity in eliciting the relaxation response. Our assumption is that self-selected music is more pleasing and familiar to patients, and that it can, therefore, increase distraction while stimulating the limbic system.

In our study, patient blinding will be impossible because the intervention will be directly linked to the type of music patients will be listening to (i.e. self-selected versus predetermined music). The outcome measure will be assessed using the most popular psychometric test in music therapy studies: the STAI questionnaire. This test has a good sensitivity to variations in anxiety [23]. Levels of anxiety will be measured at two time points, namely, before and shortly after the music-listening session. Despite randomisation, which is supposed to ensure group comparability, evaluating patient anxiety before the music-listening session seems to us necessary to avoid anxiety score imbalance.

We cannot avoid the use of different music supports: MUSIC CARE® playlists will be available only on tablets, whereas self-selected playlists will be recorded on a wide range of supports. Though the supports used may vary between the two groups, this is unlikely to affect the difference in anxiety scores and to bias our study results.

With this trial, we hope to show that the use of preferred music styles can improve the effects of music therapy. Insofar as music is a powerful, culturally specific vector of emotion, self-selected music may indeed provide an easy, low-cost, and side-effect-free alternative that can be applied anywhere. Our study may, therefore, contribute to the development of music therapy software programmes that are adapted to culturally specific contexts, as well as to the elaboration of non-medical strategies for the reduction of preoperative anxiety.

## Trial status

Enrolment began on 31 July 2017.

## Abbreviations

CRA: Clinical research associate
CRF: Case report form
NS: Numerical scale
RCT: Randomised controlled trial
STAI: Stait-Trait Anxiety Inventory
VAS: Visual analogue scale
